# Parents of children who are victims of trauma, allies or adversaries?

**DOI:** 10.1192/j.eurpsy.2023.1562

**Published:** 2023-07-19

**Authors:** H. Ben Youssef, S. Bourgou, R. Gadhoum, H. Rezgui, A. Ben Hamouda, M. Daoud, F. Charfi, A. Belhaj

**Affiliations:** 1 Child and Adolescents psychiatry, Mongi Slim Hospital, tunis, Tunisia

## Abstract

**Introduction:**

trauma affects not the child but the whole family. how would the parents’ reaction to trauma influence the child’s resilience capacities?

**Objectives:**

study the mental health status of parents of children consulting the trauma and resilience unit.

**Methods:**

Descriptive and retrospective study of 20 consultants in the trauma and resilience unit at Mongi Slim hospital between January and April 2022. The evaluation of depressive symptoms in children was made by Children's Depression Inventory (CDI). The Hamilton Depression and Anxiety Scales were used to assess anxiety and depressive symptomatology in the parents of the consultants.

**Results:**

The mean age of the children was 10.46±3.24.

The traumatic event was related to an assault in 75% of cases, 45% of which were intrafamilial, road accident and death of a relative in 10% respectively, 5% domestic violence.

Five consultants had a CDI score ≥8 and 15 had a score >10. Only parents of children with a CDI score>19 had moderate to severe symptoms according to hamilton scales.

The diagnosis of adjustment disorder was made in 45% of cases, post-traumatic stress disorder 20%, acute stress disorder 10% other 15%

**Conclusions:**

The parents’ reaction to the tragedy would play a modulating role on the children’s resilience capacities. less anxious and depressed parents would help their child build his/her coping mecanisms.

**Disclosure of Interest:**

None Declared

